# A loop-mediated isothermal amplification-enabled analytical assay for the detection of SARS-CoV-2: A review

**DOI:** 10.3389/fcimb.2022.1068015

**Published:** 2022-12-23

**Authors:** Mingna Li, Hongjuan Ge, Zhe Sun, Jangshan Fu, Lele Cao, Xinrui Feng, Guixian Meng, Yubo Peng, Yan Liu, Chen Zhao

**Affiliations:** ^1^ College of public health, Jilin Medical University, Jilin, China; ^2^ College of medical technology, Beihua University, Jilin, China; ^3^ Medical college, Yanbian University, Jilin, China; ^4^ College of medical laboratory, Jilin Medical University, Jilin, China; ^5^ Business School, The University of Adelaide, Adelaide, SA, Australia

**Keywords:** LAMP, COVID – 19, SARS-CoV-2, biosensor, technology

## Abstract

The number of words: 4645, the number of figures: 4, the number of tables: 1The outbreak of COVID-19 in December 2019 caused a global pandemic of acute respiratory disease, and with the increasing virulence of mutant strains and the number of confirmed cases, this has resulted in a tremendous threat to global public health. Therefore, an accurate diagnosis of COVID-19 is urgently needed for rapid control of SARS-CoV-2 transmission. As a new molecular biology technology, loop-mediated isothermal amplification (LAMP) has the advantages of convenient operation, speed, low cost and high sensitivity and specificity. In the past two years, rampant COVID-19 and the continuous variation in the virus strains have demanded higher requirements for the rapid detection of pathogens. Compared with conventional RT–PCR and real-time RT–PCR methods, genotyping RT-LAMP method and LAMP plus peptide nucleic acid (PNA) probe detection methods have been developed to correctly identified SARS-CoV-2 variants, which is also why LAMP technology has attracted much attention. LAMP detection technology combined with lateral flow assay, microfluidic technology and other sensing technologies can effectively enhance signals by nucleic acid amplification and help to give the resulting output in a faster, more convenient and user-friendly way. At present, LAMP plays an important role in the detection of SARS-CoV-2.

## 1 Introduction

**Figure 1:**
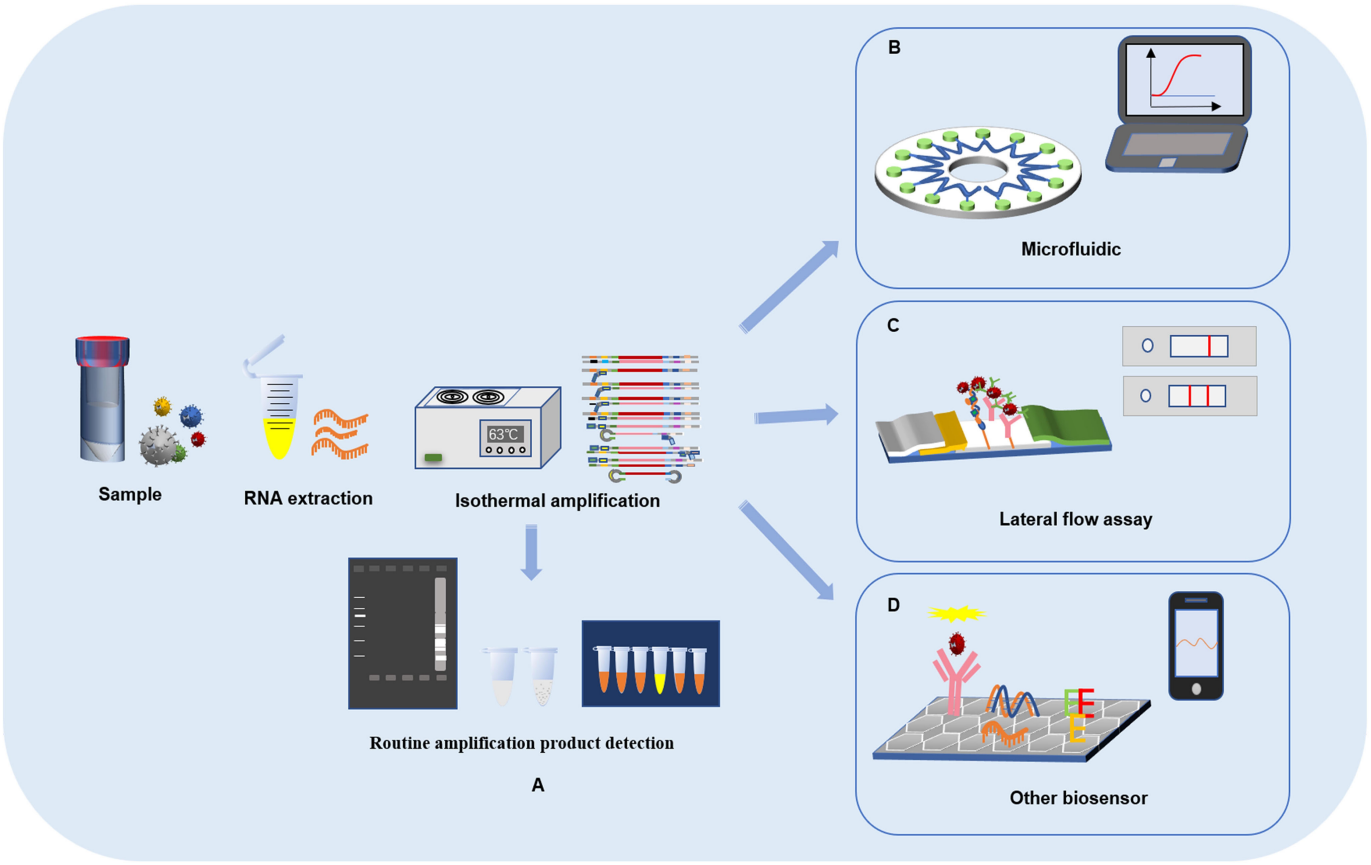
Loop-mediated isothermal amplification reaction process and detection of SARS-CoV-2 based on LAMP combined with biosensors. **(A)** LAMP reaction process and result readout. **(B)** SARS-CoV-2 detection based on LAMP combined with lateral flow assays. **(C)** SARS-CoV-2 detection based on LAMP combined with microfluidic chips. **(D)** SARS-CoV-2 detection based on LAMP combined with other biosensors.

Coronavirus disease-2019 (COVID-19), a disease that seriously threatens human life, is caused by severe acute respiratory syndrome coronavirus-2 (SARS-CoV-2). Conventionally, computed tomography (CT), immunoassay and reverse transcriptase polymerase chain reaction (RT–PCR) are used to assist in the diagnosis of COVID-19 (Pan et al., 2020; Dinnes et al., 2021; Safiabadi Tali et al., 2021; Lopera et al., 2022). The antigen load is low at the stage of infection, which makes it difficult to detect (Zhao et al., 2020). At present, SARS-CoV-2 detection mainly focuses on antibody and nucleic acid detection with quantitative reverse transcription-polymerase chain reaction (qRT–PCR) as the main method. However, these methods have a long detection time, require many reagents, and have complicated operation processes and high requirements for laboratory personnel. These disadvantages strictly restrict the rapid and accurate detection of SARS-CoV-2 for clinical screening. Due to the characteristics of isothermal amplification, which includes a short reaction time and low cost, loop-mediated isothermal amplification (LAMP) may have great potential to become an important nucleic acid detection method in SARS-CoV-2 testing. LAMP is a molecular technology for nucleic acid amplification with the characteristics of simplicity, rapidity and high specificity. The detection of SARS-CoV-2 by the LAMP method can not only rapidly and massively amplify the target fragment but also solve the limitation of PCR, which requires special instruments for changing the temperature (Yang J. et al., 2022). This review will describe the latest progress in SARS-CoV-2 detection based on LAMP combined with lateral flow assays, microfluidic technology or other biosensors and provide references for the rapid development of virus detection during the COVID-19 pandemic (**Figure 1**).

## 2 Characteristics and detection methods of SARS-CoV-2

At the end of 2019, an emerging novel coronavirus spread all over the world and caused great harm to human health. SARS-CoV-2 is a novel enveloped virus with a positive-sense, single-stranded RNA genome of ~30k nucleotides, which is closely related to SARS-CoV and has a similar clinical disease (Zhu et al., 2020). SARS-CoV-2 contains four main structural proteins embedded in the virus envelope, including spike (S), membrane (M) and envelope (E) proteins. Nucleocapsid (N) protein interacts with the virus RNA in the core of the virus particles to form the nucleocapsid and participates in the transcription and replication of virus RNA (Ning et al., 2021).

Since the initial outbreak of SARS-CoV-2 up to September 2022, the total number of confirmed cases worldwide has exceeded 600 million, with more than 6 million deaths. The virus has evolved a variety of subtypes and spread all over the world. After the virus variant VOC-202012/01 was first reported in the UK, many countries successively confirmed the infections with the Alpha (B1.1.7), Beta (B.1.351), Gamma (P.1), Delta (B.1.617.2) and Omicron (B1.1.29) mutants, which have the ability to spread rapidly (Harrington et al., 2021; Kannan et al., 2022; Kumar et al., 2022; Wink et al., 2022). According to the global initiative on sharing all influenza data (GISAID) survey, the variant rate of the S protein in SARS-CoV-2 is very high (Korber et al., 2020; Rocheleau et al., 2021). A new variant of concern (VOC) has emerged, designated as Omicron (B.1.529), which is distinct compared with the original strain (Wuhan strain) (Zhu et al., 2020), and this new variant virus has a strong infection rate (Alkhatib et al., 2022; Das et al., 2022). More importantly, viral mutations can allow the virus to evade the human immune system, and several variants display a reduction in the susceptibility to neutralization antibodies generated by natural infection or vaccination, which prolongs the epidemic period of COVID-19 (Chakraborty et al., 2022; Zhang et al., 2022). At present, China has successfully developed kits for sample collection and detection of SARS-CoV-2 based on *S*, *ORF1ab* and *N* genes with high mutation sites to identify strains that have mutated compared to the original strain (Coolen et al., 2021). The method of LAMP plus PNA probe to detect SARS-CoV-2 has been reported for the first time, and it can correctly identify the L452R spike mutation (Iijima et al., 2022). qRT–PCR is the gold standard method for detecting SARS-CoV-2 (Ford et al., 2021), but the reaction process relies heavily on temperature-changing equipment and analytical instruments. In addition, the operation process is complex and cumbersome with a long detection time, and thus it is difficult to use in remote areas and in laboratories of poor regions. LAMP is a rapid detection method with no requirement for special instruments or equipment. It is a good way to achieve early detection of SARS-CoV-2 infection to control the spread of the virus (Augustine et al., 2020).

## 3 LAMP reaction principles

The reaction process mainly includes three stages: template synthesis, cyclic amplification, and extension and recycling, resulting in a DNA mixture with multiple cauliflower and stem-loop structures. The key step of the LAMP process is the design of four primers with high specificity for the target sequence of DNA. The two inner primers are designated the forward inner primer (FIP) and backward inner primer (BIP), and the two outer primers consist of F3 and B3. The inner primer is composed of two different sequences, which correspond to the forward sequence and backward sequence of the target sequence. FIP is the upper inner primer used to initiate the reaction in the first step of template synthesis, and BIP is the lower inner primer used to carry out the second stage of cyclic amplification; the four primers participate in the LAMP reaction process in the initial stage of template synthesis, and only the inner primer is needed in the subsequent cycle reaction process. Under the action of Bst DNA polymerase, nucleic acid amplification is carried out at 60-65°C (Matthew et al., 2022). The detection of the amplified products generally includes three methods: agarose gel electrophoresis, metal ion indicator or dye coloration, and observation of the white magnesium pyrophosphate precipitate, which can be directly observed with the naked eye (Petrusha and Faizuloev, 2020).

Compared with the traditional nucleic acid amplification method, the LAMP method uses four primers for six specific regions of the target gene, which allows for a high specificity. In a clinical pathogen infection test, the limited detection of DNA or RNA copy numbers by the LAMP method is also significantly higher than that of the PCR method, and the LAMP method does not need precise instruments to control the reaction temperature, and therefore it is easy to operate. Moreover, a large number of amplification products can be obtained in a short time, and visual detection based on turbidity can be performed (Hassan et al., 2022; Wang C. et al., 2022). As a new nucleic acid amplification method, LAMP can be used not only for amplification of DNA but also for RNA amplification *via* reverse transcription. However, there will be some errors in the operation and assessment of the results by directly observing the sediments or using dye coloration after the reaction, sediments of the sample after amplification cannot remain stable for a long time. The results should be observed as soon as possible after the amplification reaction, which does not meet the standard of accurate detection (Kubota et al., 2008; Safavieh et al., 2014). Moreover, agarose gel electrophoresis for amplification products testing is usually accompanied by aerosol pollutions, which affects the accuracy of the results due to laboratory pollution. But, a facile way to rapidly configure LAMP assays by integrating OSD probes into individual and multiplex assays were demonstrated by Bhadra et al., which is an accurate probe-based readout of SARS-CoV-2 (Bhadra et al., 2021). These probes can suppressed noise from spurious amplification by LAMP primers and thereby yielded target-specific signals. An integrated modular centrifugal microfluidic platform for SARS-CoV-2 testing based on LAMP were developed by Soares et al. (2021). A few tens of virion genomic RNA could be identified by converting amplicon accumulation to color development on lateral flow dipsticks. Since agarose beads modified with dried *n*-benzyl-*n*-methylethanolamine were pre-packed in the discs to remove primer dimers selectively, the platform showed 100% specificity with fluorescence detection after the inactivation reaction. The combined application of LAMP and lateral flow assays or microfluidic technology can not only facilitate the readout of results but also effectively avoid false-positive results caused by nonspecific amplification, furtherincreasing the specificity and sensitivity of the assay.

## 4 Application of SARS-CoV-2 detection methods based on LAMP

### 4.1 RT-LAMP

Because of its convenient operation, rapid nucleic acid amplification and high specificity, RT-LAMP has been applied for the detection of SARS-CoV-2. RT-LAMP usually uses RNA as the template for amplification reactions. The viral RNA is converted into complementary DNA (cDNA) by adding reverse transcriptase to the LAMP mixture, after which the amplification reaction is carried out.

The RT-LAMP method has been used to detect SARS-CoV-2 infection, which can not only greatly shorten the reaction time but also allows for multidirectional selectivity of the target gene (Mohon et al., 2020; Urrutia-Cabrera et al., 2021). The *ORF 1ab*, *N* and *S* genes are usually used as target genes to detect SARS-CoV-2 infection (Saxena et al., 2022; Talap et al., 2022). A one-step RT-LAMP method for detecting SARS-CoV-2 was developed and evaluated (Park et al., 2020). The primers of the one-step RT-LAMP method were designed with the *S* and *N* genes of SARS-CoV-2, the reaction conditions were optimized, and the reaction mixture was detected by using colorless crystal violet with a colorimetric detection method. The assay can be completed within 30 min from the amplification reaction to the detection of the fluorescence signal. A RT-LAMP method targeting the *ORF 1ab* and *N* genes was established and carried out under isothermal conditions of 63°C from reverse transcription to result read-out (Wang Y. et al., 2021). Fourteen copies/reactions of SARS-CoV-2 were detected in 35 min.

A small amount of viral RNA can be amplified by RT-LAMP that increases the detection rate of the virus. And a variety of samples can be used for nucleic acid detection, among which saliva, nasopharyngeal swabs and alveolar lavage fluid are samples that are currently used to detect SARS-CoV-2 nucleic acid (Kitsou et al., 2021; Kundrod et al., 2022). RT-LAMP and qRT–PCR methods were used to assess the control sample and viral RNA extracted from the patient’s nasopharyngeal swab, and the specificity of RT-LAMP was evaluated (Lamb et al., 2020). The results showed that SARS-CoV-2 could be specifically detected in clinical patient samples within 30-40 min. Compared with qRT–PCR, the RT-LAMP method may allow for faster and cheaper field-based testing at the point of risk. In addition, a rapid colorimetric RT-LAMP method was developed to amplify nasopharyngeal swab samples from SARS-CoV-2-infected patients after high-temperature treatment (Dao et al., 2020). It was found that the structure of viral RNA was more stable after short-term high-temperature treatment. The RT-LAMP method for detection of SARS-CoV-2 samples had a high accuracy, and thus the researcher proved the feasibility of RT-LAMP in the on-site testing of SARS-CoV-2. RT-LAMP plays an important role in the detection of SARS-CoV-2, which is very helpful for the diagnosis of COVID-19 and on-site screening at the port of entry.

### 4.2 LAMP combined with the lateral flow assay

**Figure 2:**
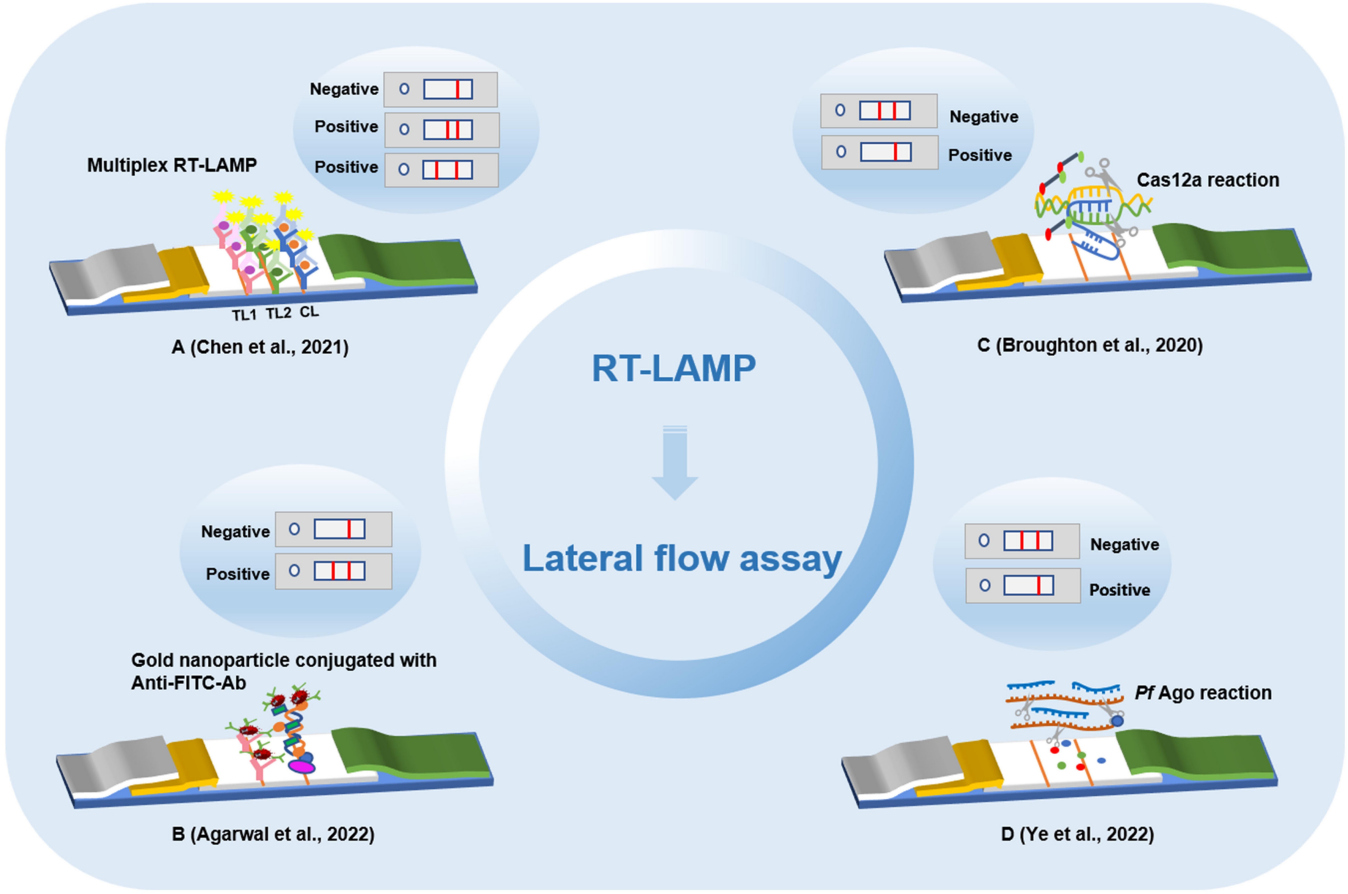
Detection method of lateral flow assay based on loop-mediated isothermal amplification technology. **(A)** Multiplex reverse transcription loop-mediated isothermal amplification linked with gold nanoparticle-based lateral flow assay. **(B)** Lateral flow assay with enzyme incorporation of biotin labeled dUTP. **(C)** Lateral flow assay based on loop-mediated isothermal amplification and Cas12a. **(D)** Lateral flow analysis of Argonaute integrated loop-mediated isothermal amplification.

The lateral flow assay (LFA) uses a nitrocellulose membrane as the carrier. The sample solution is added dropwise on the sample pad, where it permeates and moves toward the end of the absorption pad under the action of a capillary. The target binds to the receptor, and fluorescent and quantum dot markers are used to detect the optical reaction so that the signal value can be detected on the test line or control line. LFA has the characteristics of portability, low cost and efficiency, which have allowed it to become an ideal choice for point-of-care testing (POCT), and has been widely used in the rapid detection of various targets, such as bacteria, viruses, parasites, mycotoxins, and with the continuous innovation and development of LFA technology, a photothermal test strip assay that combines test strips with a portable photothermal card reader was established for the sensitive, rapid and quantitative detection of residues of food hazards (Wang et al., 2020; Charlermroj et al., 2021; Wang, X. et al., 2021; Wang Y. et al., 2022b; Yang J. et al., 2022). An LFA based on LAMP to identify tissue of cattle origin has been developed with high specificity and sensitivity (Jawla et al., 2021). The components of LAMP reaction were lyophilized over test strip, a pair of probes was designed, tagged and its hybridization with the amplified product of LAMP reaction was optimized. This method were eliminated the lengthy DNA extraction step and the detection results were shown to be completely consistent with the PCR assay. RT-LAMP combined with LFA for rapid and accurate detection of zika virus was proposed (Ahn et al., 2021). This method is based on LFA reaction, hybridization occurred between the AuNPs: polyadenylated (polyA10) -ZIKV probe and the LAMP amplicons. The results can be detected in less than 5 min and reduce the number of false positives. A rapid antigen detection kit based on LFA has been widely used during the COVID-19 outbreak. China has developed and approved LFAs for the detection of SARS-CoV-2 infection, and IgM and IgG antibodies or antigens of SARS-CoV-2 in patients can be detected within 15 min (Li et al., 2021; Liu et al., 2021). When the disease is at an early stage, focusing on antibodies or antigens testing may lead to false negative results. With the development of nanotechnology, carbon nanoparticles and carbon nanotubes have signaficantly improved the sensitivity of LFA detection results, and nucleic acid detection is the gold standard for the diagnosis of new coronary pneumonia. At present, the method of LAMP combined with LFA to detect SARS-CoV-2 has been gradually proposed by many scholars (Zasada et al., 2020; Jang et al., 2021; Zheng et al., 2021) (**Figure 2**).

After the biotin-labeled LAMP product is hybridized with the FITC-labeled specific probe and the FITC-labeled specific probe is combined with the anti-FITC antibody on gold nanoparticles, the immune complex is added to the test strip. When the immune complex diffuses through the chromatographic membrane to the detection line, the biotin-labeled amplification product is captured by the biotin ligand and develops color. The combined application of the two methods can be effectively used for the detection of SARS-COV-2 with the naked eye. A new method for the combined application of nanoparticle-based flow test strips and RT-LAMP has been established (Chen et al., 2021). The method can be used for tests of the *RdRp* and *N* genes of SARS-CoV-2, and the whole reaction process can be completed in only 1.5 h with 100% specificity and the limit of detection was 20 copies/reaction. A method combining RT-LAMP with LFA was proposed for detecting SARS-CoV-2 (Agarwal et al., 2022). In this method, biotin and FITC were combined with 11 dUTP and LF primers on the strip to produce highly specific results. The results can be detected in 15 min with high accuracy. CRISPR-Cas12 based gene editing technology has also been applied to the detection of SARS-CoV-2 infection. After extracting the viral RNA from the throat swab sample, RT-LAMP amplification was carried out. Cas12 targeted the predetermined viral nucleic acid sequence. Then, a reporter molecule was cleaved to confirm the presence of virus nucleotides, and the results were interpreted in strips. The method of detecting SARS-CoV-2 based on CRISPR-Cas12, which combines LAMP and LFA, can quickly detect SARS-CoV-2 from samples. Compared with qRT–PCR, this method has reliable accuracy and was verified to be feasible (Broughton et al., 2020). A novel DNA capture probe-based LFA for detecting SARS-CoV-2 was proposed (Yi et al., 2021). First, based on a CRISPR/Cas12a detection method, a biotinylated reporter gene was designed according to the *S* and *N* genes of SARS-CoV-2. Second, the RT-LAMP assay was used to improve reaction sensitivity. In addition, a customized reporter was used, which was hybridized with an optimized complementary capture probe fixed on the test line for result readout through two independent systems: strips for the naked eye and real-time fluorescence signals. The combined application of RT-LAMP and LFA to detect SARS-CoV-2 can achieve ultrasensitivity of 1 copy/μL within 60 min. This will provide strong support for the detection of SARS-CoV-2 and other pathogens in laboratories in poor or remote areas. To break the limitation of the complexity and instability of multiple detection caused by guide RNA in the CRISPR-based method, an integrated nucleic acid detection system (Mulan) integrating Argonaute (Ago) and RT-LAMP was established (Ye et al., 2022). The system combines the multiple inclusiveness of RT-LAMP and a single Ago molecule, which can specifically detect a variety of pathogens, including SARS-CoV-2 and influenza A and B viruses, and analyze the results in the form of flow test strips and fluorescent signals. This method can alleviate the shortage of false-positive from LAMP detection by Argonaute’s specifically cleave target directed by base-pairing guide.

### 4.3 LAMP combined with microfluidic technology

**Figure 3:**
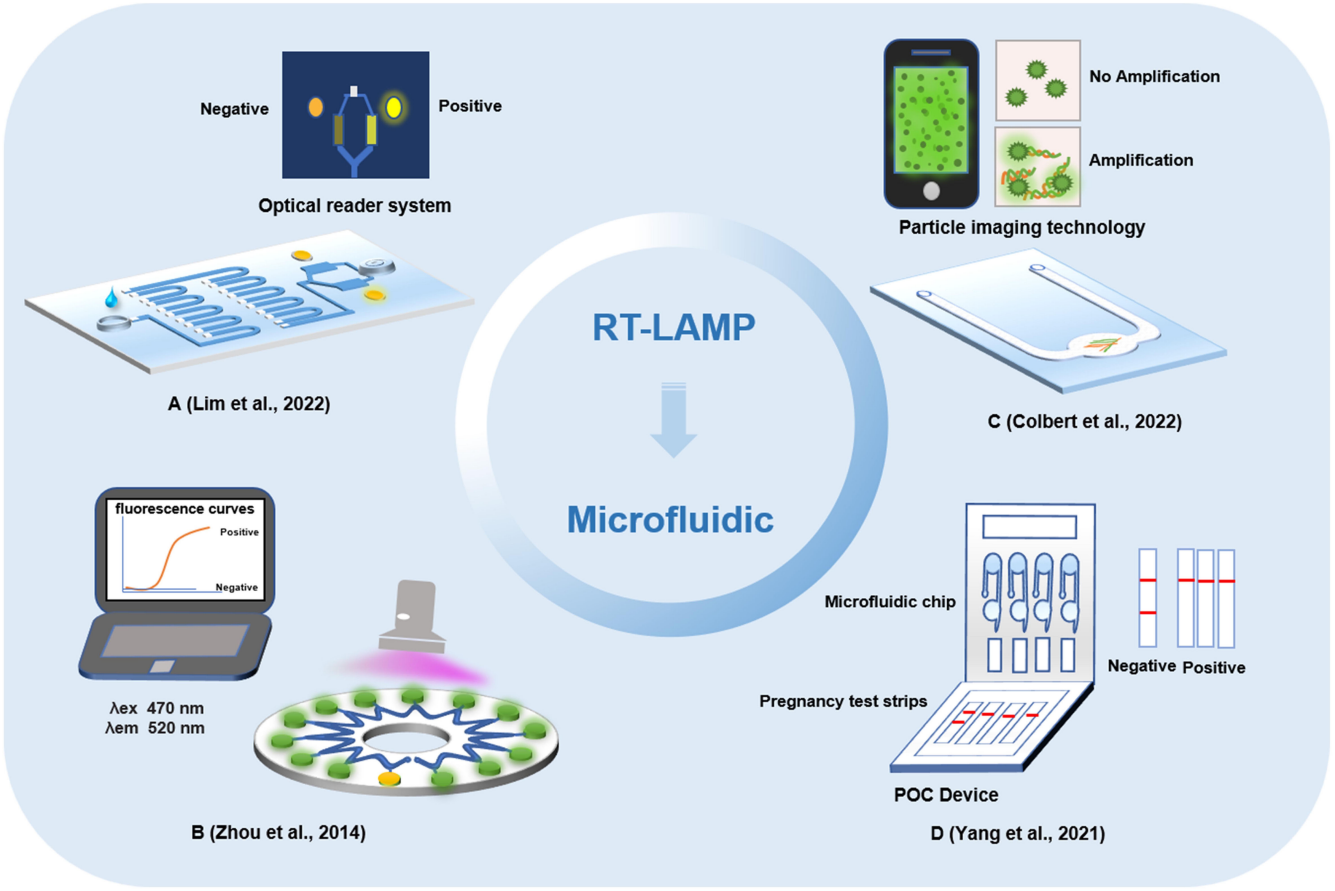
Detection method of microfluidic chips based on loop-mediated isothermal amplification technology. **(A)** Microfluidic point-of-care device integrated plastic cartridges. **(B)** Real-time fluorogenic loop-mediated isothermal amplification assay integrated on a microfluidic disc chip. **(C)** Portable microfluidic chip based on particle imaging technique, particle diffusometry. **(D)** Microfluidic device based on commercial pregnancy test strips and a palm size.

As the core technology of a micrototal analysis system, microfluidic analysis integrates chemical, electronic, computer and other function modules on one chip to establish biochemical analysis by controlling the flow of trace liquid; it has the characteristics of small sample requirement, controllability, robust function, and rapid and sensitive detection (Yuan et al., 2019). The development of a lab-on-chip (LOC) system based on the integration of LAMP and microfluidic technology is expected to speed the detection of pathogens and allow the early diagnosis of diseases (**Figure 3**). Currently, LAMP technology on LOC has been applied to the detection of breast cancer biomarkers, dengue serotypes, carbapenemase-producing organisms, and multiple pathogens in the lower reproductive tract during pregnancy (Moser et al., 2022; Wormald et al., 2022; Wu et al., 2022; Xu et al., 2022). Microfluidic chips are characterized by high throughput and low cost, a method of LAMP technology combined with a microfluidic chip to detect a variety of pathogens has been established, which can perform detection of multiple pathogens and real-time detection within 40 min without aerosol pollution and cross reaction (Ou et al., 2021). Research on the combined application of LAMP technology and microfluidic chips to detect pathogens is of great significance for the rapid detection of SARS-CoV-2 infection.

A microfluidic analysis and equipment for detecting the SARS-CoV-2 alpha mutant based on the RT-LAMP method were demonstrated. SARS-CoV-2 was successfully detected in the saliva of patients by using the *N* and *S* genes (Lim et al., 2022). At the same time, this method can also distinguish the alpha mutant from the early variant of SARS-CoV-2. The method of LAMP combined with a microfluidic chip mainly loads the nucleic acid template to the inlet the of microfluidic chip, with 10 copies/μL of targets detected within 0.5 h. Subsequently, based on the joint application of LAMP and microfluidic chips, the research team developed a multiple virus nucleic acid detection kit (Zhou et al., 2014). This kit can detect a variety of pathogens, including influenza A virus and SARS-CoV-2, showing high discrimination performance. A method for detecting SARS-CoV-2 based on RT-LAMP and particle imaging technology, particle diffusometry (PD), was designed to detect virus particles by isothermal amplification on an integrated heated portable chip, and then smartphone devices were used for fluorescence imaging and particle diffusion rate analysis. With this method, SARS-CoV-2 could be detected in only 35 min. This method has a high specificity and sensitivity, and the potential of cross contamination can be eliminated (Colbert et al., 2022). To further improve the portability of the combined application of LAMP and microfluidic chips, an on-site, semiautomatic detection system was developed (Yang M. et al., 2022). The entire detection was integrated into a four-channel, palm-size microfluidic device. SARS-CoV-2 can be detected within 2 h by detecting the RNA signal generated by isothermal amplification and then reading the results with a portable commercial pregnancy test strip. It provides a rapid, cost-effective, and sensitive assay, with a limit of detection at 0.5 copy/μL for SARS-CoV-2 RNA.

### 4.4 LAMP combined with other biosensors

**Figure 4:**
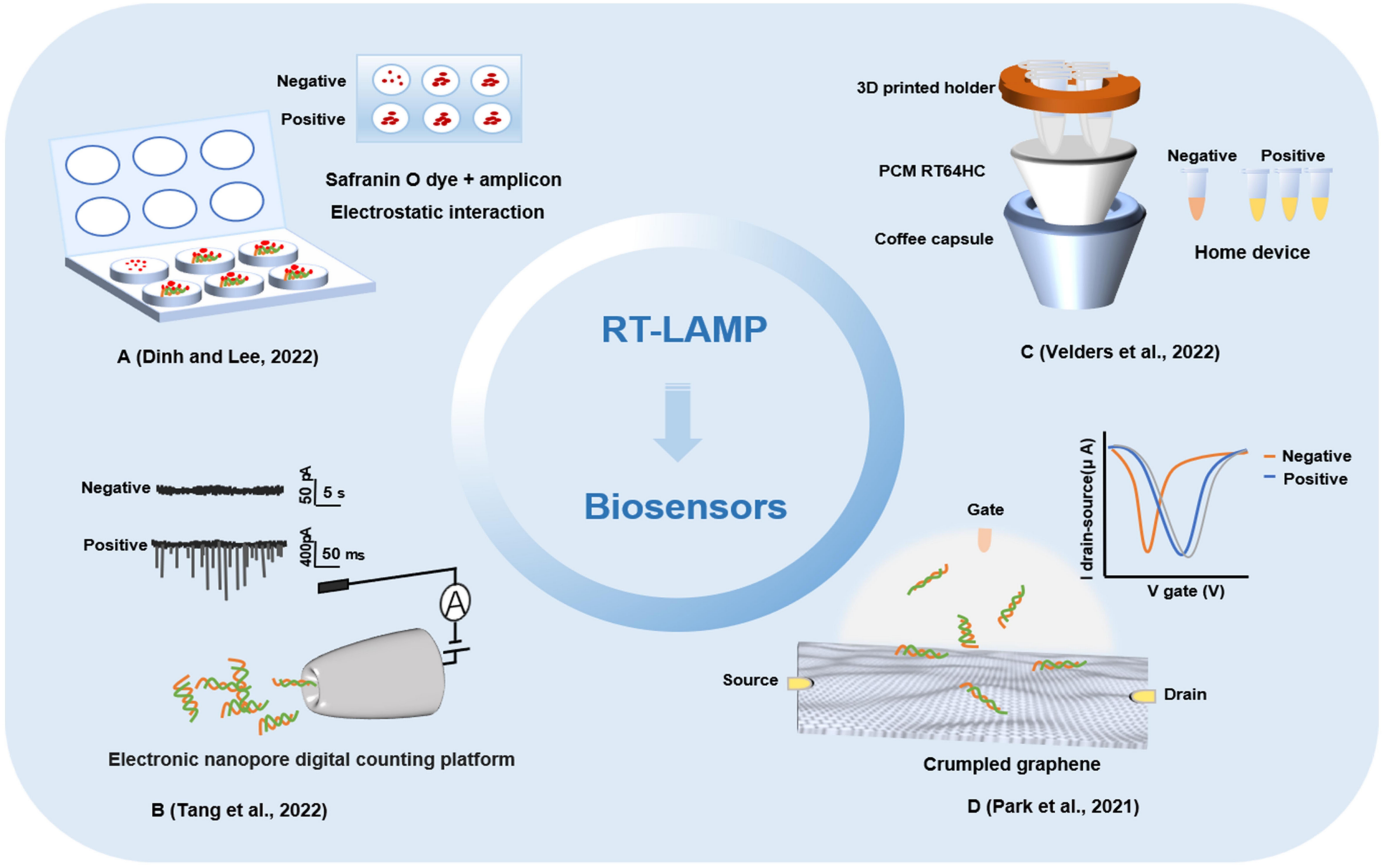
Other biosensors detection methods based on loop-mediated isothermal amplification technology. **(A)** A fully integrated paper microdevice of using Safranin O dye coupled with loop-mediated isothermal amplification. **(B)** Reverse transcription loop-mediated isothermal amplification coupled glass nanopore digital counting method. **(C)** T-Cup: A cheap, rapid, and simple home device for isothermal nucleic acid amplification. **(D)** A crumpled graphene field-effect transistor biosensor.

The biosensor is composed of a molecular recognition part and a conversion part, and the measured substance can be sensed by the sensitive element and then transformed into an identifiable signal output. In recent years, electrochemical biosensors, optical biosensors, and colorimetric biosensors have been widely used in pathogen detection (Mahshid et al., 2021; Pinals et al., 2021; Lin et al., 2022; Park et al., 2022). Due to the fast analysis speed, high sensitivity and low price of biosensor technology, it has been widely used and adapted for new applications by researchers involved in virus detection, disease screening and diagnosis in laboratories. During the COVID-19 outbreak, a variety of biosensors were developed to detect SARS-CoV-2 infection. A biosensor device based on a field-effect transistor (FET) was used to detect SARS-CoV-2 in samples (Seo et al., 2020). The biosensor device was coated with a specific antibody against the SARS-CoV-2 S protein through the graphene in the FET. As a highly sensitive method for the diagnosis of COVID-19, the biosensor successfully detected SARS-CoV-2 in the sample, with a limit of detection at 2.42 x 10^2^ copies/mL for clinical samples. The electrochemical biosensor based on FET has the advantages of low cost, high sensitivity and wide dynamic response range. It provides a highly sensitive diagnostic method for clinical detection of SARS-CoV-2 without any pretreatment or labeling of samples. In addition, an FET biosensor based on graphene oxide graphene (GO/Gr) van der Waals heterostructures has been developed (Gao et al., 2022). The GO/Gr van der Waals heterostructure was *in-situ* formed in the microfluidic channel through π-π stacking. GO with abundant functional groups (OH-, COOH-, CO-) has an improved adsorption force for target molecules than graphene, and the abundant functional groups of GO nanosheets reacted strongly with SARS-CoV-2 capture antibodies *via* both π–π stacking and hydrogen bonding. This method can not only detect SARS-CoV-2 within 20 min but also has strong selectivity and sensitivity, providing a potential method for fast and accurate SARS-CoV-2 detection. At present, a piezoelectric microcantilever biosensor, a vertical microcavity and localized surface plasmon resonance hybrid biosensor and an electronic labeling strategy of protein molecules, and demonstration of a SARS-CoV-2 protein biosensor employing a colloidal quantum dot (CQD)-modified electrode have all been proposed (Kabir et al., 2021; Zhao et al., 2022; Zheng et al., 2022). These biosensor devices mostly utilize antigens or antibodies for biological recognition. Recently, various detection methods for nucleic acid-based biosensor devices have been developed (**Figure 4**).

Combining RT-LAMP with a glass nanopore biosensor, a detection method for SARS-CoV-2 was demonstrated with a high specificity, sensitivity, and portability and rapid operation (Tang et al., 2022). The RNA of SARS-CoV-2 virus was reverse transcribed and amplified by RT-LAMP, and then the concentration of products was measured by nanopore sensor and digital counting methods. The optimized RT-LAMP assay targeting the N gene showed the limit of detection was 65 copies and possessed an excellent specificity. With its integration capability, the biggest advantage of the nanopore digital analysis method is that it has the characteristic of single molecule level sensitivity and can read and analyze the process of RT-LAMP-amplified nucleic acid more quickly. A paper microdevice including nucleic acid extraction, amplification and signal reading was developed (Dinh and Lee, 2022). The device utilizes the electrostatic interaction between a negatively charged LAMP amplicon and positively charged saffron O oligomers to effectively detect SARS-CoV-2 through colorimetry. In comparison with the existing fully integrated devices, this strategy could reduce the cost and reaction time by using eco-friendly paper and naked-eye detection. And this method will be able to directly detect SARS-CoV-2-positive samples on site without the requiring specialized instruments. A method for the rapid detection of SARS-CoV-2 by LAMP combined with a nanoparticle biosensor (RT-LAMP-NBS) was established (Suleman et al., 2021). Two sets of RT-LAMP primers were designed according to the *ORF1ab* and *N* genes to specifically identify 8 regions of the target gene. In the specific analysis, the positive control and samples of SARS-CoV-2 were positive results, while noninfected samples showed no false-positive results. In the clinical diagnosis of patients with COVID-19, RT-LAMP-NBS has 100% sensitivity and specificity for SARS-CoV-2 samples, and the time from sample collection to result is greatly shortened. Research shows that RT-LAMP-NBS is an effective means to diagnose SARS-CoV-2 infection and can be applied for robust clinical infection detection (Seo et al., 2020). With the continuous innovation of detection methods for SARS-CoV-2, home devices and POCT have become the mainstream development trend of virus detection. A home temperature cup (T-Cup) device based on LAMP for testing SARS-CoV-2 was created by using a simple aluminum coffee capsule, a phase change material (PCM) and a 3D printed holder (Velders et al., 2022). The paraffin-based PCM and 3D printed vial holder were placed in the cup together with the PCR tubes. PCM can be melted when it reaches the melting point in a hot water bath and maintained at a constant temperature of 61-67°C for 25 min, which meets the conditions required for the LAMP reaction. Finally, the colorimetric method was used to observe the results. T-Cup and PCR were used to detect positive and negative samples of SARS-CoV-2 infection, and the results of the two methods were consistent, which shows that T-Cup has a certain feasibility in detecting SARS-CoV-2 infection. The development of biosensors and their combined application with LAMP technology has provided a foundation for the rapid detection of SARS-CoV-2 (**Table 1**).

**Table 1:** Detection of SARS-CoV-2 based on different nucleic acid amplification strategies.

Nucleic acid amplification strategy	LOD	Specificity	Time (min)	Instrument	Ref
RT-PCR	100 copies/reaction	100%	-^*^	Heavy	(Carvalho et al., 2021)
	-^*^	100%	-^*^	Heavy	(Linssen et al., 2022)
	-^*^	100%	135	Heavy	(Dorlass et al., 2020)
Real time -PCR	10 copies/reaction	100%	250	Heavy	(Klein et al., 2020)
	5 copies/μL	100%	90	Heavy	(Gibani et al., 2020)
	1 copies/μL	100%	-^*^	Heavy	(Wu et al., 2020)
RT-LAMP	8 copies/reaction	95%	30	Portable	(Ooi et al., 2022)
	1.5 copies/μL	-^*^	30-50	Portable	(Huang et al., 2021)
	0.2 copies/μL	-^*^	40	Portable	(He et al., 2021)
LAMP-LFA	-^*^	100%	100	Portable	(Zhu et al., 2021)
	1.9 copies/μL	-^*^	105	Portable	(Wang Y. et al., 2022a)
	1.0 copies/μL	100%	60	Portable	(Ge et al., 2022)
LAMP-Microfluidic	2x10^2^ copies/μL	-^*^	90	Portable	(Jhou et al., 2022)
	200 copies/μL	-^*^	30	Portable	(Natsuhara et al., 2021)
	10 copies/μL	100%	60	Portable	(Soares et al., 2021)
LAMP-Other biosensor	0.2 copies/mL	99.5%	-^*^	Portable	(Ptasinska et al., 2021)
	10 copies/μL	100%	60	Portable	(Park et al., 2021)
	50 copies/μL	100%	40	Portable	(Ganguli et al., 2020)

*: No test.

## 5 Conclusion

As an isothermal nucleic acid amplification method, LAMP has been pursued as an ideal low-tech alternative for rapid and portable testing, a large number of studies for promoting the transformation of LAMP technology to POCT have showed great potentials in clinical (Mautner et al., 2020). The LAMP based biosensors to detect SARS-CoV-2 infections has great advantages, but there are still some shortcomings and challenges. First, false positive detection results caused by aerosol pollution in nucleic acid amplifications have attracted extensive attentions of researchers. Microfluidic chip provide micro closed channels and micro reaction chambers with different shapes and sizes for LAMP (Zhang et al., 2019). At the same time, the multi-channel LAMP diagnostic integrated system can make the micro reaction chambers to be connected in parallel without interference (Peng et al., 2019). In addition, the results can be directly detected after the reaction in the chip, without exposing to the environment. Therefore, the combined application of LAMP and microfluidic technology effectively overcomes the disadvantage of traditional LAMP caused by aerosol pollution. Secondly, LAMP requires a minimum of four primers to target six binding sites with strict requirements regarding the distances between each of the binding sites while each primer must also meet specific conditions. The complicated LAMP primer design makes primer development time-consuming. Once a set of reliable primers is developed, the sensitivity and amplification rate of LAMP will be further improved and does not produce non-specific amplification. Finally, most of signal out-put after nucleic acid amplifications are fluorescence or color changes during the reaction with subjective judgments. The current technology development and research mainly focus on how to complete the quantitative detection with rapid, high specificity and sensitivity without exposing the amplified products, which is an inevitable trend in the development of nucleic acid detection and POCT.

Currently, the number of cases of COVID-19 and asymptomatic patients worldwide is still rising, and developments and innovations in detection methods for SARS-CoV-2 are also ongoing to control the rapid spread of the epidemic. As an effective assay with a high specificity and sensitivity, convenient operation and low price, LAMP can be used for the robust detection of SARS-CoV-2 nucleic acid when combined with a variety of biosensors, which make up for the shortcomings of traditional PCR detection technology including equipment dependence and long detection cycles, so that nucleic acid detection can be performed in a wide range of environments or locations. The combined application of LAMP technology with other biosensors for the detection of SARS-CoV-2 has great advantages, and rapid and accurate detection for identifying SARS-CoV-2 is of great significance for disease prevention and control.

## Author contributions

ML: Conceptualization, Methodology, Writing – original draft, Investigation, Visualization. HG: Methodology, Investigation, Visualization, Writing – review & editing.SZ, JF, LC, XF, GM, YP: Investigation, Visualization, Writing – review & editing. YL: Conceptualization, Methodology, Visualization, Supervision, Writing – review & editing. CZ: Funding acquisition, Conceptualization, Methodology, Supervision, Visualization, Writing – review & editing. All authors contributed to the article and approved the submitted version.
